# Use of electroanalgesia and laser therapies as alternatives to opioids for acute and chronic pain management

**DOI:** 10.12688/f1000research.12324.1

**Published:** 2017-12-21

**Authors:** Paul F. White, Ofelia Loani Elvir Lazo, Lidia Galeas, Xuezhao Cao

**Affiliations:** 1 P.O. Box 548, Gualala, CA 95445, USA; 2 The White Mountain Institute, The Sea Ranch, CA, USA; 3 Department of Anesthesiology, Cedars-Sinai Medical Center, 8700 Beverly Boulevard, Los Angeles, CA 95445, USA; 4 Orlando Family Medicine, Orlando, FL, USA; 5 First Hospital of China Medical University, Shenyang, China

**Keywords:** Non-pharmacologic analgesic techniques, Opioid abuse, Acute Pain, Chronic Pain, Electroanalgesia, Cold laser therapy

## Abstract

The use of opioid analgesics for postoperative pain management has contributed to the global opioid epidemic. It was recently reported that prescription opioid analgesic use often continued after major joint replacement surgery even though patients were no longer experiencing joint pain. The use of epidural local analgesia for perioperative pain management was not found to be protective against persistent opioid use in a large cohort of opioid-naïve patients undergoing abdominal surgery. In a retrospective study involving over 390,000 outpatients more than 66 years of age who underwent minor ambulatory surgery procedures, patients receiving a prescription opioid analgesic within 7 days of discharge were 44% more likely to continue using opioids 1 year after surgery. In a review of 11 million patients undergoing elective surgery from 2002 to 2011, both opioid overdoses and opioid dependence were found to be increasing over time. Opioid-dependent surgical patients were more likely to experience postoperative pulmonary complications, require longer hospital stays, and increase costs to the health-care system. The Centers for Disease Control and Prevention emphasized the importance of finding alternatives to opioid medication for treating pain. In the new clinical practice guidelines for back pain, the authors endorsed the use of non-pharmacologic therapies. However, one of the more widely used non-pharmacologic treatments for chronic pain (namely radiofrequency ablation therapy) was recently reported to have no clinical benefit. Therefore, this clinical commentary will review evidence in the peer-reviewed literature supporting the use of electroanalgesia and laser therapies for treating acute pain, cervical (neck) pain, low back pain, persistent post-surgical pain after spine surgery (“failed back syndrome”), major joint replacements, and abdominal surgery as well as other common chronic pain syndromes (for example, myofascial pain, peripheral neuropathic pain, fibromyalgia, degenerative joint disease/osteoarthritis, and migraine headaches).

## Introduction

 Pain is a common cause of disability and is extremely costly to society at large. The excessive reliance on opioid analgesics for treating both acute and chronic pain has contributed to the current global opioid epidemic. Recently, the Centers for Disease Control and Prevention (CDCP) reported that patients initially given a 1-day supply of opioid medication after surgery had a 6% likelihood of still using the drug a year later. However, that number rose to roughly 10% for patients given a 2-day supply and 45% for patients given a 40-day prescription. In light of the growing opioid crisis, we have emphasized the importance of using non-opioid analgesic drugs and techniques for acute and chronic pain management ^1– 3^.

 In 2007, a review article by international experts in pain management encouraged the more widespread use of opioid-containing analgesics for treating chronic pain by suggesting that “if only we (namely, physicians and nurses) could overcome our ‘opiophobia’, we would improve pain management” ^4^. In an accompanying editorial ^1^, we argued that “less may be more” with respect to the use of opioid (narcotic) analgesics for acute (and chronic) pain therapy. Currently, 5–8 million people in the USA use opioids for chronic pain management ^5^. It has become increasingly apparent that prolonged opioid use is a growing problem in previously opioid-naïve patients who receive narcotics for acute pain after a surgical procedure. Excessive reliance on opioid analgesics for postoperative pain control can lead to prolonged opioid dependence after both major ^6^ and minor ^7^ surgery. In a large retrospective study involving over 390,000 older outpatients undergoing minor ambulatory surgery procedures ^7^, older patients receiving prescription opioid analgesics within 7 days of discharge were 44% more likely to continue using opioids 1 year after surgery. More recently, Goesling *et al.* ^6^ reported that for many patients taking opioid analgesics before major joint replacement surgery, as well as opioid-naïve patients undergoing arthroplasty procedures, opioid use persisted after surgery despite the absence of joint-related pain. More recently, Cauley *et al.* ^8^ reported on a large cohort of opioid-naïve patients undergoing abdominal surgery who continued to use opioid analgesics 3–6 months after surgery. Moreover, adjunctive use of epidural local analgesia was not protective against persistent opioid use in this opioid-naïve population ^9^. Although opioid-related side effects (for example, nausea, vomiting, constipation, ileus, bladder dysfunction, pruritus, sedation, visual hallucinations, and ventilatory depression) are well known, there are growing concerns regarding long-term physical dependence and addiction liability with continued opioid use after surgical procedures.

Prolonged use of opioid analgesics is associated with an increased risk of more serious complications, including opioid use disorder, overdose, and death ^10^. Interestingly, no study of prolonged opioid use has ever demonstrated long-term benefits for the users. An epidemiological study by Eriksen *et al.* ^11^ involving patients with chronic pain treated with opioids for 5 years provided compelling evidence that opioids were not a panacea for chronic pain. In fact, the patients’ quality of life failed to improve despite escalating doses of opioids over the 5-year study period. The authors concluded that “it is remarkable that opioid treatment of long-term/chronic non-cancer pain does not seem to fulfil any of the key outcome treatment goals, namely pain relief, improved quality of life and improved functional capacity” ^11^. More recent studies suggest that long-term opioid use can actually retard functional recovery. In a 2012 commentary, Sullivan and Ballantyne ^12^ asked the rhetorical question, “what are we treating with long-term opioid therapy?” As the opioid epidemic continues to spread and deaths due to opioid overdoses increase, it is clear that the only “winners” are the pharmaceutical drug manufacturers.

A 2016 article in *Time* magazine described a new paradigm for treating opioid addiction ^13^. Predictably, the solution being offered by the pharmaceutical industry involves giving more drugs! The “new” drug touted as the solution to opioid dependency (Suboxone) is a combination of two old drugs, namely buprenorphine (a weak partial opioid agonist) and naloxone (an opioid antagonist). A more recent article ^14^ describes the cost of the “follow on” drugs used to counteract opioid-related side effects (for example, nausea, constipation, and reduced testosterone levels) and overdoses. In 2016, US pharmaceutical companies had sales of $8.6 billion on 336 million opioid prescriptions. The “follow on” drug market is currently valued at more than $3 billion per year, and sales of drugs used to counteract opioid-induced constipation exceed $2 billion per year ^14^. It is time to seriously consider the use of “alternative” non-pharmacologic therapies for treating chronic pain rather than simply giving more opioid compounds and hoping for a different result ^3^.

Given the fact that the long-term use of opioid analgesics fails to improve the lives of patients suffering from chronic pain and costs the health-care system billions of dollars to treat opioid overdoses, it is somewhat surprising that the health-care practitioners (and third-party payers) have been reluctant to seriously consider the use of non-pharmacologic “alternative” therapies for managing pain. The use of non-pharmacologic modalities has been questioned by medical practitioners because of the perceived lack of prospective, randomized, double-blind sham-controlled studies supporting their use in clinical practice. Although this skepticism may be warranted for some so-called alternative pain therapies, there are in fact many well-controlled studies confirming the benefits of using electroanalgesia and laser therapy for improving acute and chronic pain management. Nevertheless, these non-pharmacologic analgesic modalities remain grossly under-utilized in clinical practice. In a 2006 clinical commentary regarding a study involving the use of complementary alternative therapies for reducing postoperative pain after open heart surgery ^15^, Oz and Olivo ^16^ suggested that it was time to consider incorporating complementary and alternative medical practices into conventional medical treatments. Other studies have suggested that perioperative imagery, massage, and music can reduce pain and anxiety after surgery ^17, 18^. A 2007 editorial on the potentially beneficial role of “alternative” therapies as part of a multimodal approach for reducing anxiety, pain, and emetic symptoms in the perioperative period ^19^ suggested that it was time for practitioners to “get on board” by incorporating non-traditional medical therapies into their everyday clinical practices. Sadly, 10 years later in the face of an opioid epidemic, the emphasis of the medical community remains on pharmacotherapy. In this F1000 Faculty Review, we discuss clinical studies describing the benefits of using electroanalgesia and laser therapy as adjuvants in the management of acute and chronic pain.

## Electroanalgesia

Electroanalgesia is a form of neuromodulation therapy which encompasses electro-acupuncture (EA), ultrasound-guided acupotomy, percutaneous electrical nerve stimulation (PENS), transcutaneous electrical nerve stimulation (TENS), and peripheral nerve stimulation (PNS). Patients who experience significant improvement in pain and disability with PNS may be candidates for implantation of a spinal cord stimulator ^20, 21^. However, the risk of mechanical failure, infection, and neurologic complications, as well as the high costs, are strong deterrents to using this highly invasive electro-analgesic technique. The less invasive forms of electroanalgesia (for example, TENS, EA, PENS, and PNS) have been reported to produce significant short-term reductions in the levels of acute and chronic pain and should be used prior to even considering the use of an implantable device.

Gan *et al.* ^22^ reported that TENS applied at acupoints reduced both postoperative pain and emesis, confirming earlier studies ^23– 27^. TENS also reduced pain and improved patient satisfaction during minor office procedures ^28^. However, in a comparison of TENS and parasternal local anesthetic blocks for pain management after cardiac surgery ^29^, the local anesthetic-based technique was found to be significantly more efficacious. TENS produced modest short-term reductions in pain and improvements in physical activity in patients experiencing a variety of chronic pain syndromes (for example, low back pain ^30– 35^, neck pain ^36^, osteoarthritis/gonarthritis ^37, 38^, abdominal/pelvic pain ^39^, myofascial pain syndrome ^40^, and temporal-mandibular disorders ^41^). However, a recent review of electrotherapy modalities for treating chronic rotator cuff diseases ^42^ concluded that, in contrast to more invasive electroanalgesia modalities (for example, EA), TENS was not consistently superior to placebo treatments. In patients undergoing total knee arthroplasty procedures, TENS offered no significant advantage with respect to improving pain control and functional recovery compared with placebo treatments ^43^. Finally, Salazar *et al.* ^44^ recently reported that there was only low-quality evidence supporting the effectiveness of TENS for pain relief in patients with fibromyalgia. However, these investigators found moderate-quality evidence for the effectiveness of EA in treating fibromyalgia-related pain ^44^. Thus, the current evidence suggests that TENS techniques are significantly less effective than electroanalgesia techniques such as EA and PENS, which involve the insertion of multiple acupuncture-like needles ^45^.

Sator-Katzenschlager *et al.* ^31^ and others ^32^ have reported on the short- and long-term benefits of EA in patients with chronic low back pain. In a recent study involving elderly patients undergoing spine surgery, Zhang *et al.* ^46^ found that preoperative EA (versus “sham” treatments) reduced both intraoperative anesthetic and analgesic requirements, levels of inflammatory mediators, and residual postoperative cognitive dysfunction in elderly surgical patients. These EA studies are consistent with studies involving the use of PENS in patients with chronic low back pain ^47^ and sciatica ^33^. Moreover, PENS was found to be significantly more effective than TENS and exercise therapy ^33, 47^. In a recent comparative study, PENS was found to be more effective than “dry needling” in improving pain control in patients with myofascial chronic neck pain ^48^. A growing body of literature supports the short-term benefits of EA and PENS techniques when administered as an adjuvant to conventional medical approaches in the management of a wide variety of acute and chronic pain syndromes, including post-surgical pain, low back pain, sciatica, neck pain, knee osteoarthritis, headaches, peripheral neuropathic pain, and fibromyalgia ^33, 44, 47– 52^. In a recent comparative study, EA was also found to be more effective in facilitating functional recovery in patients with knee osteoarthritis than the potent non-steroidal anti-inflammatory drug meloxicam ^53^. Interestingly, acupotomy EA was reported to be superior to conventional EA in patients with knee osteoarthritis with respect to pain control and functional recovery ^54^. Despite numerous controlled studies in the peer-reviewed literature supporting their clinical efficacy ( Table 1), minimally invasive electroanalgesia techniques have failed to gain widespread clinical acceptance because they are time-consuming to perform and insurance reimbursement is extremely low compared with other medical procedures. Although there is some ‘cumulative’ benefit after a series of electroanalgesia treatments, maintenance therapy is typically required because the duration of pain relief after each treatment session is fairly short-lived (<48 hours).

**Table 1.** Comparison of typical characteristics and applications of transcutaneous and percutaneous electroanalgesia techniques for treating acute and chronic pain.

	Transcutaneous electroanalgesia techniques	Percutaneous electroanalgesia techniques
Examples	Transcutaneous electrical nerve stimulation (TENS) Acupoint-like transcutaneous electrical nerve stimulation	Electroacupuncture (EA) Percutaneous electrical nerve stimulation (PENS) Peripheral nerve stimulation
Application	Non-invasive Cutaneous pads (disposable)	Minimally invasive Acupuncture needles inserted through skin
Duration of pain relief	Limited short-term benefits (<24 hours)	Short-term and some longer-term benefits (24–72 hours)
Cost	Low (self-administered)	High (personnel required)
Time required to perform techniques	Minimal	More labor-intensive and time-consuming to perform
Applications for acute and chronic pain management	Acute postoperative pain, low back pain, neck pain, osteoarthritis/gonarthritis, abdominal/pelvic pain, myofascial pain syndrome, chronic rotator cuff diseases, and temporomandibular disorders	Acute post-surgical pain, low back pain, sciatica, neck pain, knee osteoarthritis, osteoarthritis/ gonarthritis, headaches, peripheral neuropathic pain, and fibromyalgia

## Laser therapy

Compared with electroanalgesia and exercise therapy, laser therapy is a relatively recent development in medicine. The first cold laser was US Food and Drug Administration (FDA)-approved for treating pain in 2001, and low-level laser therapy (LLLT), also known as cold laser therapy, has been used in the USA since only 2002. High-intensity laser therapy (HILT), also known as laser heat therapy, is an even more recent development; initial publications appeared in 2011. Laser therapy involves a simple, non-invasive, “point-and-shoot” technique which can be performed by technicians. Cellular chromophores are presumed to be the receptor sites responsible for the beneficial effects of the laser light beam, including both cytochrome c oxidase (with absorption peaks in the near-infrared range) and photoactive porphyrins ^55^. Mitochondria are also thought to be a site for the therapeutic effects of infrared light, leading to increased ATP production, modulation of reactive oxygen species, and induction of transcription factors. These effects lead to increased cell proliferation and migration by fibroblasts; reduction in the levels of cytokines, growth factors, and inflammatory mediators; and increased tissue oxygenation, leading to enhanced control of the inflammatory process, reduced pain, and improved wound healing ^56– 59^. Studies with laser therapy have confirmed enhanced wound healing in both diabetic ^60^ and non-diabetic ^59, 61, 62^ patients. Applying LLLT within the first 5 days of herpes zoster eruption also significantly reduced the incidence of post-herpetic neuralgia ^63^.

Many sham-controlled studies have reported that LLLT is effective in alleviating acute pain associated with a variety of superficial medical and surgical conditions (for example, oral surgery ^64– 66^, nipple pain associated with prolonged breastfeeding ^67^, plantar fasciitis ^68, 69^, and carpel tunnel syndrome ^70^). In women with chronic myofascial pain, LLLT reduced the pain intensity ^71^. Interestingly, LLLT has been reported to reduce acute pain after coronary artery bypass graft surgery ^72, 73^. Recent studies also reported that LLLT could improve sensory function in patients with peripheral somatosensory neuropathy and neuropathic pain ^74, 75^. However, other investigators have reported sham-controlled studies with negative results using LLLT in patients with superficial medical conditions (for example, plantar fasciitis) ^76^. Despite early clinical studies reporting benefits from LLLT in patients with fibromyalgia ^77, 78^, a recent study by Vayvay *et al.* ^79^ reported that LLLT was not significantly better than sham treatments and Kinesio Tape.

Several studies have described the use of LLLT for treating dental pain after oral surgery, with reduced orthodontic pain at 6 hours, 24 hours, 3 days, and even 1 week after surgery ^80^. Alan *et al.* ^81^ reported that although LLLT reduced trismus and swelling after oral surgery, pain was only decreased on the 7th postoperative day. More recently, LLLT administered after maxillofacial surgery was reported to accelerate healing and enhance quality of life related to oral health ^82^. However, Chen *et al.* ^83^ reported that LLLT had limited efficacy in reducing pain in patients with temporal-mandibular joint disorders. Although LLLT is reported to be more effective than “traditional procedures” in the management of oro-facial pain, it remains to be determined which power level and wavelength produce the optimal outcomes ^84^.

LLLT has been reported to be a useful adjunct treatment for oral mucositis in patients with cancer ^85– 90^. However, in patients with breast cancer and unilateral lymphedema, LLLT failed to significantly improve their quality of life, pain scores, grip strength, or limb volume ^91^. In contrast, long-term beneficial effects have been reported with pulsed HILT in the treatment of post-mastectomy pain syndrome ^92^. A recent literature review ^93^ suggested that LLLT might be a promising option for the management of cancer treatment-related side effects (for example, oral mucositis, radiodermatitis, lymphedema, and chemotherapy-induced peripheral neuropathic pain). Although LLLT has been reported to have a suppressive effect on cancer cells ^94– 97^, studies using rodent models suggest that it might modify cancer cell behavior and actually lead to stimulation of dysplastic cells ^98– 101^. For example, Rhee *et al.* ^102^ reported that LLLT increased tumor size in rodents when thyroid cancer cells were directly exposed to photodynamic (laser) therapy. Clearly, additional studies with both LLLT and HILT are needed in patients with cancer.

In a sham-controlled, prospective safety and efficacy study of LLLT in patients with subacute musculoskeletal back pain, Basford *et al.* ^103^ concluded that LLLT produced a “moderate” reduction in pain while improving the patients’ perception of clinical benefit and level of functionality. Glazov *et al.* ^104^ found evidence to support a short-term benefit of LLLT in treating low back pain in patients with a shorter duration of pain symptoms. However, these investigators suggested that greater pain-relieving benefits were achieved when higher laser dosages were administered (that is, HILT versus LLLT). This speculation was confirmed by Boyraz *et al.* ^105^ and Chen *et al.* ^106^, who reported that HILT in combination with exercise produced significant functional improvements with longer-lasting beneficial effects than exercise therapy alone in patients with lumbar disc herniation. HILT also appeared to be an effective alternative to spine surgery for reducing pain and improving the performance of activities of daily living in patients with chronic back pain ^107^.

In a recent review ^42^, the authors stated that LLLT has only short-term benefits (versus sham) in treating rotator cuff disease; however, HILT was effective in minimizing pain and disability and increasing range of motion in patients with shoulder pain ^108^. Similarly, in a 2013 systematic review of LLLT in treating chronic neck pain, the authors reported that although the benefits observed were statistically significant, the differences failed to achieve a “minimally-important clinical difference” ^109^. In contrast, Alayat *et al.* found that HILT combined with exercise in patients with chronic low back ^110^ and neck ^111^ pain was more effective than exercise therapy alone and also had a more sustained effect in decreasing pain and functional disability (for example, improving range of motion), and beneficial effects lasted for up to 3 months. Chow *et al.* ^112^ reported that laser therapy reduced pain immediately after treatment sessions in patients with acute neck pain and for up to 22 weeks after completion of a series of treatments in patients with chronic neck pain. Haładaj *et al.* ^113^ demonstrated that HILT also produced clinically-significant analgesic efficacy in patients with cervical radicular pain syndrome and cervical spondylosis. Dundar *et al.* ^114^ reported that HILT is an effective therapeutic method in the treatment of patients with myofascial pain syndrome of the trapezius muscle, a common cause of chronic neck pain.

Kim *et al.* ^115^ and others ^116^ reported that HILT was an effective non-surgical intervention for patients with knee osteoarthritis, reducing pain and improving their ability to perform activities of daily living. Alayat *et al.* ^117^ reported that, in men with osteopenia and osteoporosis, HILT combined with exercise was more effective than exercise alone in reducing pain and improving quality of life after a series of treatments. Kheshie *et al.* ^118^ and others ^119^ have reported that HILT is significantly more effective than LLLT in treating chronic osteoarthritis-related pain. These findings with HILT (versus LLLT) were also confirmed in a study by Alayat *et al.* ^120^ in patients with Bell’s palsy. HILT was also effective in the short-term management of pain and disability related to subacromial impingement syndrome, frozen shoulder and lateral epicondylitis ^121– 123^. HILT at wavelengths of 830 and 1,064 nm was better than TENS in improving control of pain and paresthesias secondary to carpal tunnel syndrome ^124^. Analogous to LLLT, HILT has also been used to treat acute headache pain ^125– 127^, degenerative joint conditions ^115– 119, 128– 131^, neuropathic pain syndromes ^64, 132– 136^, and a wide variety of musculoskeletal disorders, including fibromyalgia ^77– 79, 114, 137^.

A more powerful version of HILT has been introduced using class IV lasers producing more than 25 W of power (for example, the Phoenix Thera-Lase device) ( Table 2). Huang *et al.* ^138^ demonstrated that the beneficial outcomes of laser therapy were directly related to the amount of energy (power) administered. The energy density (in joules per square centimeter) or power (in watts) appears to be strongly related to the efficiency of laser radiation in reducing pain and inflammation. Power density represents the laser/light-emitting diode incident power in watts on the tissue spot size (that is, output power divided by the size of the irradiated area in watts per square meter). The higher power density is responsible for regulating or “speeding up” the transport of electrons in the mitochondrial respiratory chain ^90^. The World Association of Laser Therapy has established that target tissues need an energy density of 5–7 J/cm ^2^ or higher to elicit a clinically significant biological cellular response ^139^.

**Table 2.** Comparison of primary characteristics of low-level laser therapy and high-intensity laser therapy devices for the management of common pain syndromes.

	Low-level laser therapy (LLLT)	High-intensity laser therapy (HILT)
Laser class	I, Im, II, III	IV
Wavelength	600–980 nm	660–1,275 nm
Power	<1 W	1–75 W
Penetration abilities	Low (<2 cm)	Deep (5–15 cm)
Temperature changes	<1.0°C	Low thermal accumulation
Applications for acute and chronic pain management	Superficial postoperative pain Osteoarthritis Low back pain Neck pain Plantar fasciitis Dental pain Mucositis-associated pain Acute and chronic pain related to herpes virus Trigeminal neuralgia Wound repair The pain of muscle injury Shoulder pain Carpal tunnel syndrome Fibromyalgia-related pain Headache Opioid dependency	Postoperative pain Osteoarthritis Hemophilic arthropathy Low back pain related to herpes virus Myofascial pain syndrome Shoulder pain Fibromyalgia-related pain Opioid dependency Note: Several of the applications listed for low-level laser therapy have not been studied using high-intensity laser therapy devices. However, there is no reason to expect that they would not respond as well or better.

**Figure 1.**
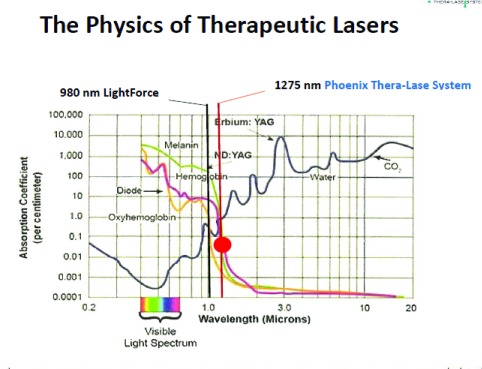
The absorption effect of different wavelengths of infrared light by water, hemoglobin, oxyhemoglobin, and melanin. This figure illustrates one of the effects of the wavelength differences between the Phoenix Thera-Lase and other commercially available high-intensity laser therapy devices (for example, LightForce/LiteCure, erbium-doped yttrium aluminium garnet laser [Er:YAG], and neodymium-doped YAG [Nd:YAG]) with respect to infrared light absorption. These spectra are available from online sources.

The more powerful HILT devices are also referred to as laser heat devices ^140^. In addition, some of these powerful laser devices function at longer wavelengths; for example, the Phoenix Thera-Lase (Phoenix Thera-Lase Systems, Dallas, TX, USA), which produces up to 75 W of power, operates at a wavelength of 1,275 nm. Therefore, the laser beam produced by these devices can penetrate more deeply into the soft tissue because of their enhanced power and the reduced absorption of the laser beam by melanin and hemoglobin ( Figure 1) ^3^. Profound and long-lasting analgesic effects have recently been reported with this HILT device in patients with chronic post-surgical pain requiring the use of opioid analgesics ^141^, professional athletes with degenerative joint disease requiring non-opioid analgesics ^142^, and a woman with long-standing drug-resistant fibromyalgia ^137^. The fibromyalgia case report suggests that the use of HILT at 42–75 W of power can produce more profound and longer-lasting beneficial effects than standard treatment protocols utilizing LLLT ^79^ and less powerful HILT devices ^140^. Importantly, LLLT studies have reported that photobiomodulation therapy produced greater improvement in muscular performance and accelerating recovery when it is administered prior to exercising ^143, 144^. Although Foley *et al.* ^145^ reported that LLLT facilitated a faster recovery of injured university athletes, they failed to include either a control or an active comparator group.

Larger-scale studies are clearly needed to verify the benefits of HILT compared with both LLLT and electroanalgesia in decreasing acute and chronic pain and improving long-term clinical outcomes. Finally, determining the optimal power and wavelength for each clinical condition is critically important in optimizing both LLLT and HILT. For example, de Oliveria *et al.* ^144^ compared 100, 200, and 400 mW of power and found that with LLLT a higher power does not necessarily produce a better result with respect to improvement in muscle performance. These authors also recommended using a wavelength of 810 nm for achieving optimal results with LLLT. However, Bordvik *et al.* ^146^ reported that a 910 nm wavelength produced greater tissue penetration than 810 nm with a super-pulsed laser.

Super-pulsed class IIIB lasers (for example, Multi Radiance, Medical [Solon, Ohio], and Thera-Lase [Toronto, Ontario, Canada]) can produce a peak power of up to 100 W without causing tissue warming in contrast to the more powerful class IV lasers (for example, LightForce lasers [LiteCure, Newark, DE] produces 0.5–25 W at wavelengths of 980/810 nm and Phoenix Thera-Lase [Phoenix Thera-Lase Systems, Dallas, TX] produces 1–75 W at a wavelength of 1,275 nm). In a sham-controlled study, Leal-Junior and colleagues ^147^ reported that a super-pulsed laser was effective in decreasing pain and improving the quality of life in patients with non-specific knee pain. Of interest, a recent study by de Marchi *et al.* ^148^ reported that low-powered ‘pulsed’ lasers produced superior effects with respect to delayed-onset muscle soreness and elevations in creatine kinase activity than both low- and high-powered continuous lasers. Notarnicola *et al.* ^149^ reported that HILT with 5 W of power at wavelengths of 650 nm, 810 nm, and TRIAX (810/980/1,064 nm) reduces low back pain scores and disability at 1, 2, and 4 months post-treatment. The greatest efficacy in promoting nerve regeneration and modulating pain transmission was associated with the use of the longer wavelengths (810/980/1,064 nm versus 650 nm).

## Discussion

In a recent “call to action” to end the opioid epidemic in this country ^150^, the US Surgeon General pointed out that the annual number of overdose deaths involving prescription opioids has nearly quadrupled since 2000 and this increase parallels the marked growth in the quantity of opioid pain relievers being prescribed ^151^. Prescription opioid addiction and misuse have also contributed to a resurgence in heroin use and the spread of HIV and hepatitis C ^152^. In the “Turn the Tide” Rx Pocket card which the government recently mailed to all practicing clinicians in this country, there was no mention of non-pharmacologic approaches to treating pain. However, in the recent FDA response to the opioid crisis, Califf *et al.* ^153^ emphasized that more “alternatives” to opioid analgesics are needed, including non-pharmacologic therapies. According to the FDA leadership, “non-pharmacologic approaches to pain treatment were identified as an urgent priority” ^153^. The new Clinical Guidelines Committee of the American College of Physicians also endorsed the use of non-invasive treatments such as laser therapy for the treatment of acute, subacute, and chronic low back pain ^154^. The CDCP has also recently emphasized the importance of finding non-pharmacologic alternatives to opioid analgesic medications for treating pain ^155^. These authors reported that patients initially given a 1-day supply of opioid medication had a 6% likelihood of still using the drug a year later. However, that number rose to roughly 10% for patients given a 2-day supply and to 45% for patients given a 40-day prescription.

Of importance, Barnett *et al.* ^156^, in a recent issue of the *New England Journal of Medicine*, reported that long-term opioid use was increased in previously opioid-naïve patients who received treatment in an emergency department from high (versus low) intensity opioid prescribers. Altering the prescribing habits of physicians and their surrogates will clearly help; however, in order to effectively deal with the current opioid crisis, more innovative non-pharmacologic approaches for treating acute and chronic pain are clearly needed ^157^. In this review, we have described numerous clinical studies published within the last 5 years which support the use of non-pharmacologic electro-analgesic and laser therapies for managing both acute and chronic pain.

## Conclusions

The consequences of the widespread reliance on opioid-containing medication for managing acute and chronic pain should not be surprising. Many different electro-analgesic and laser therapy techniques have been described in the peer-reviewed literature for treating acute and chronic pain. However, these “alternative” therapies have failed to achieve broad acceptance in the medical community because of lingering questions regarding their analgesic efficacy, a lack of long-term outcome studies, and low reimbursement by third-party payers to the health-care providers administering these non-traditional therapeutic modalities. Although the use of powerful HILT devices appears to produce more profound and sustained beneficial effects than electroanalgesia and LLLT in the treatment of acute and chronic pain, this therapeutic modality has also failed to achieve widespread acceptance in the medical community because of a lack of knowledge regarding the potential long-term benefits of HILT among health-care providers, low third-party reimbursement rates, and the high cost associated with purchasing these more powerful laser devices. The mechanism of action of both electroanalgesia and laser therapy appears to involve neuromodulation of peripheral nerves using low-level electrical currents ^158^ and infrared light ^159, 160^, respectively. Acustimulation, a popular form of electroanalgesia, has been reported to trigger the release of neurotransmitters and endogenous opioid-like substances and to activate c-fos within the central nervous system (CNS). Laser photobiomodulation appears to induce direct inhibitory effects on peripheral nerves, which reduces acute pain input into the CNS. In chronic pain patients, laser-induced changes in the spinal cord produces longer-term suppression of pain in the CNS.

Given the current opioid crisis in the USA, it is time to seriously consider incorporating these “alternative” analgesic therapies into treatment protocols for managing acute and chronic pain rather than adding more “fuel to the fire” by administering even more opioid-related drugs (157). A simple, safe, and effective non-invasive pain therapy without side effects could significantly reduce the dependence on oral opioid-containing medications in the post-discharge period after surgery. These non-pharmacologic therapies would also be cost-effective alternatives to opioids for treating chronic pain not responding to non-opioid analgesic medications. It would appear that both electroanalgesia and laser therapy are safer and more cost-effective for managing chronic pain than the long-term use of opioid analgesics. Finally, HILT could prove to be a valuable therapy for treating patients who have become addicted to opioid-containing prescription medications.
